# Contentment

**Published:** 1886-12-18

**Authors:** 


### Words of Consolation.

[Communications for this column should be addressed, "Chaplain," care of the Editor of The Hospital.]

### XII.—Contentment.

Perhaps the virtue at the heading of this article should have been treated somewhat earlier in the series. If a sufferer is to be patient, hopeful, cheerful, he must have withal a contented mind. "Godliness with contentment is great gain" (1 Tim. vi, 6). And surely, the more difficult the circumstances under which contentment is preserved the greater the gain. "I have learnt in whatsoever state I am therewith to be content" (Phil. iv, 2). We never really know what our godliness is worth until it has been tested under various states. To be contented in health is one thing, but in sickness quite another. Did not Satan show a deep knowledge of human nature in reserving until the last the sore disease with which he was allowed to afflict Job? Job proved faithful throughout the whole of his fiery trials; but it is easy to see that the earlier stages of his tribulation, the loss of servants, property, even children, aroused no such terrible temptation to discontent as the plague in the flesh. "Only put forth Thine hand and touch his bone and his flesh, and he will curse Thee to Thy face" (Job ii, 5). Alas! how many of us have found it so. We have sustained other losses with tolerable equanimity, bereavements of relations or of friends very dear to us; or pecuniary embarrassments have been made the best of without much complaining. But, at last, when the very flesh has been touched, we have found ourselves, if not quite cursing our lot, murmuring, complaining, and embittered in spirit as we never were before. How ashamed we have been to have to own such selfishness, at least to ourselves. To think we could have been fairly contented under other reverses or trials which, perhaps, affected the persons of our relatives more than our own, and then to have failed in sickness because it has touched the very self in the flesh! What a humiliating thought! To feel, again, that our religion, upon which we rather prided ourselves, was not so much wanting under those other trying circumstances, and yet should have forfeited much of its sustaining power in sickness, because, at last, it was robbed of contentment. This was sad. We do not, however, make such reflections with any desire of rendering some already saddened heart sadder. No, we only want to point out the remedies for this grievous pest of discontent. Briefly, they are as follows:—

1. You must believe discontent is not less than sin. You have been sorely tried; you are suffering still. But do not make your suffering or your circumstances any excuse for further indulgence of the evil spirit.

2. Consider it your constant duty to fight against this evil spirit. But you cannot fight alone. Ask God for help the instant you feel discontented thoughts arising about anything—let us say, over food, medicines, the treatment of attendants, or the conduct of other patients.

3. Do not be out of heart if you make slow progress. You think your lot bad: but who placed you in your present position? Is it not written, "The lot is cast into the lap; but the *whole* disposing thereof is of the Lord" (Prov. xvi, 33).

4. Remember, however much you are called to endure, your Lord and Saviour was contented to bear more for you.

(*To be continued.*)

